# Supplementary material for: Motor cortex analogue neurons in songbirds utilize Kv3 channels to generate ultranarrow spikes
Source: eLife. 2023 May 9;12:e81992. doi: 10.7554/eLife.81992 (PMC10241522; doi:10.7554/eLife.81992)
Supplement: Supplementary file 1. — Geometrical properties and spine densities measured from the reconstructions of the neuronal morphologies. Values of the axon area include only the visible portions connected to the soma. Axons excluded from volume measurements. [file elife-81992-supp1.docx]

|  | RA1 | RA2 | RA3 | AId1 | AId2 |
| --- | --- | --- | --- | --- | --- |
| Soma area (µm^2^) | 1125 | 599 | 915 | 721 | 733 |
| Dendrite area (µm^2^) | 5219 | 5672 | 4252 | 4143 | 6953 |
| Spine area (µm^2^) | 530 | 923 | 463 | 827 | 1074 |
| Axon area (µm^2^) | N/A | 99 | 286 | N/A | 1264 |
| Total area (µm^2^) | 6874 | 7194 | 5630 | 5691 | 8760 |
| Total Spine # | 401 | 517 | 276 | 850 | 974 |
| Spines/ µm | 0.25 | 0.28 | 0.2 | 0.45 | 0.39 |
| Dendritic Length | 1588 | 1883 | 1414 | 1901 | 2504 |
| Total volume (µm^3^) | 4722.6 | 3155.5 | 3737.6 | 2301.2 | 3017.8 |
| Total area/volume (1/µm) | 1.456 | 2.280 | 1.506 | 2.473 | 2.903 |
